# Modulation of pro-apoptotic effects and mitochondrial potential on B16F10 cells by DODAC/PHO-S liposomes

**DOI:** 10.1186/s13104-018-3170-7

**Published:** 2018-02-14

**Authors:** Arthur Cássio de Lima Luna, José Roberto de Assis Santos Filho, Henrique Hesse, Salvador Claro Neto, Gilberto Orivaldo Chierice, Durvanei Augusto Maria

**Affiliations:** 1 Biochemistry and Biophysical Laboratory, Butantan Institute, 1500, Vital Brasil Avenue, Sao Paulo, 05503-900 Brazil; 2 Department of Medical Sciences, Medical School, University of Sao Paulo, Sao Paulo, Brazil; 3 Department of Chemistry and Molecular Physics, University of Sao Paulo, Sao Carlos, Brazil

**Keywords:** Liposomes, Nanotechnology, Melanoma, Phosphoethanolamine

## Abstract

**Objective:**

We aimed to evaluate the potential of DODAC/PHO-S liposomes on the modulation of the expression of pro-apoptotic proteins, loss of lysosomal integrity and the mitochondrial electrical potential, compared with phosphoethanolamine.

**Results:**

The results of this study demonstrate that DODAC/PHO-S liposomes have exhibited broad cytotoxic potential in B16F10 murine melanoma cells, with significantly greater proportions than treatment with PHO-S. The treatment with the DODAC/PHO-S 2.0 mM liposomal formulation was more efficient in decreasing mitochondrial electrical potential at the same concentrations and treatment time than PHO-S The liposomal formulation DODAC/PHO-S (2.0 mM) was more efficient to promote morphological changes in the cells, without presenting intact lysosomes, at the same time of treatment and concentration as PHO-S Our results demonstrated that the liposomal formulation increased DR4 receptor expression and activated caspases 8 and 3, resulting in the release of cytochrome c in B16F10 tumour cells, when compared to treatment with PHO-S The data obtained prove that the use of DODAC as carrier can maximize the cytotoxic effects of PHO-S This was demonstrated by the translocation of cytochrome c to the cytoplasm and activation of caspase-3 and 8, decreasing the mitochondrial electrical potential and generating morphological changes, in B16F10 cells.

## Introduction

Taking into account the limiting factors of the therapies available for the treatment of melanoma, new treatments that are more effective and less harmful are necessary. Thus, synthetic phosphoethanolamine (PHO-S), a phosphoric ester known as amino-ethyl phosphoric ester, has previously been synthesized by our group. Our results demonstrated that PHO-S was cytotoxic on melanoma tumor cells associated with apoptotic effect, without any effect on normal cells, as such human normal endothelial, fibroblasts and lymphocytes cells [1–3]. However, its cytotoxic effect and selectivity against tumor cells could increase with encapsulation in cationic liposomes, such as dioctadecyldimethylammonium chloride (DODAC), due to electrostatic interactions between these liposomes and tumor cell membranes [4].

Recently our group encapsulated PHO-S in DODAC liposomes (DODAC/PHO-S), because this cationic liposomes can interact with the PHO-S due to electrostatic attraction, in aqueous solution after being subjected to phase transition temperature and subsequent sonication. Physical–chemical analysis showed that these liposomes were stable [4].

The treatment of B16F10 and Hepa1c1c7 cells with DODAC/PHO-S liposomes demonstrated the lowest values of IC_50%_ for tumour cells, compared with PHO-S alone, with an IC_50%_ of 0.8 mM for B16F10 cells and 0.2 mM for Hepa1c1c7 cells, and without significant effects on endothelial cells [4]. Thus, we aimed to evaluate the potential of DODAC/PHO-S liposomes on modulates the expression of pro-apoptotic proteins and the mitochondrial electrical potential.

## Main text

### Materials and methods

#### Liposomal formulation DODAC/PHO-S

Synthetic phosphoethanolamine was synthesised in accordance with the previous studies [1–3, 5–8]. Posteriorly, DODAC (Sigma, St. Louis, MO, EUA) powder was weighed and suspended in 10 mL of water, in order to obtain final concentrations of 0.3; 0.6; 1.0; 1.3; 1.6; 2.0 mM. Further, PHO-S was included in the solution, at the same concentration of DODAC (DODAC:PHO-S = 1/1 molar ratio). The dispersions were maintained at 57 °C, for 20 min., until complete homogenization. Samples were vortexed and sonicated using a Braun Sonic 1510 ultrasonic apparatus (Branson, EUA) equipped with a titanium tip (70 watts, 3–4 min, at 60 °C). After sonication, the samples were centrifuged at 1500 rpm for 5 min to remove residual titanium released from the probe. The liposomes were sterilised by filtration through a 0.22 µm Millipore filter [1–9].

#### Cell culture

B16F10 murine melanoma cells (ATCC^®^ CRL-6475) was cultured in RPMI medium (LGC Biotecnologia, Cotia, SP, Brazil). Medium was supplemented with 2 mM l-glutamine (Cultilab, Campinas, SP, Brazil), 10 mM HEPES (Cultilab, Campinas, SP, Brazil), 24 mM sodium bicarbonate, 0.01% of antibiotics and 10% fetal bovine serum (Cultilab, Campinas, SP, Brazil). Cells were cultivated in 5% CO_2_ atmosphere at 37 °C as monolayers cultures. Cells were checked for viability using the Trypan Blue exclusion test.

#### Analysis of changes in mitochondrial electrical potential (ΔmΨ) and lysosomal integrity by confocal laser scanning microscopy

The analysis of the mitochondrial potential was realized by confocal laser scanning microscopy. B16F10 cells were plated on sterile glass slides in 24-well plates, at a concentration of 10^5^. After 24 h, the cells were treated with PHO-S and DODAC/PHO-S at 0.3 and 2.0 mM, for 6 h. Then, the cells were washed three times with culture medium RMPI (Cultilab, Campinas, SP, Brazil) without supplementation, at 37 °C and incubated with 15 µg/mL of rhodamine-123 (Assay Designs Inc., Ann Arbor, MI, USA), for 10 min. in the dark, at 37 °C. The excess rhodamine-123 was washed with culture medium at 4 °C. For lysosomal integrity assay, the cells were incubated with 10 µg/mL of acridine orange (Molecular Probes Inc., Eugene, OR, USA), for 10 min. in the dark, at 37 °C. The excess of fluorochrome was washed with culture medium at 37 °C. The analysis was realized by confocal laser scanning microscopy (Carl Zeiss LSM 700; Leica, Mannheim, Germany).

#### Analysis of expressions of markers by flow cytometry

The B16F10 cells (1 × 10^5^ cells/well, the cell density reached 80–90% confluence), treated with PHO-S (2.0 mM), DODAC/PHO-S 1:1 (0.3–2.0 mM), and empty DODAC (0.3–2.0 mM), for 12 h, were washed with PBS and re-suspended in FACS buffer with 2.5% paraformaldehyde for 1 h. The cell membrane was permeabilized in 0.1% Triton ×100 for 30 min. on ice. Cells were washed and re-suspended in FACS buffer. After washing, cells were re-suspended in a primary antibody anti-cytochrome c (ab13575, Abcam, Cambridge, MA, United States), anti-caspase-8 (sc70501, Santa Cruz Biotechnology Inc, Santa Cruz, EUA) and anti-caspase-3 (sc7272, Santa Cruz Biotechnology Inc, Santa Cruz, EUA) and anti-DR4 (ab9809, Abcam, Cambridge, MA, United States) at a concentration of 1 μg/mL at 4 °C, for 1 min. The corresponding isotope antibody was used as negative control and as a secondary antibody: Goat anti Mouse IgG (H/L): Ficoeritrina–Invitrogen (Invitrogen, Carlsbad, EUA) was used. The analysis were performed on BD Biosciences FACs Calibur flow cytometer (Becton–Dickinson, San Jose, CA, United States) using Cell Quest software.

#### Statistical analysis

All values were expressed as mean ± standard deviation (SD). Each value is the mean of at least three independent experiments in each group. One-way analysis of variance (ANOVA) and Tukey–Kramer multiple comparisons test was performed. Graphics were obtained by Prism version 5.0 (CEO and Founder, La Jolla, CA, USA) software. *P* values < 0.05, < 0.01, and < 0.001 are statistically significant.

## Results

### DODAC/PHO-S liposomes increase PHO-S cytotoxicity in B16F10 cells

**Fig. 1:**
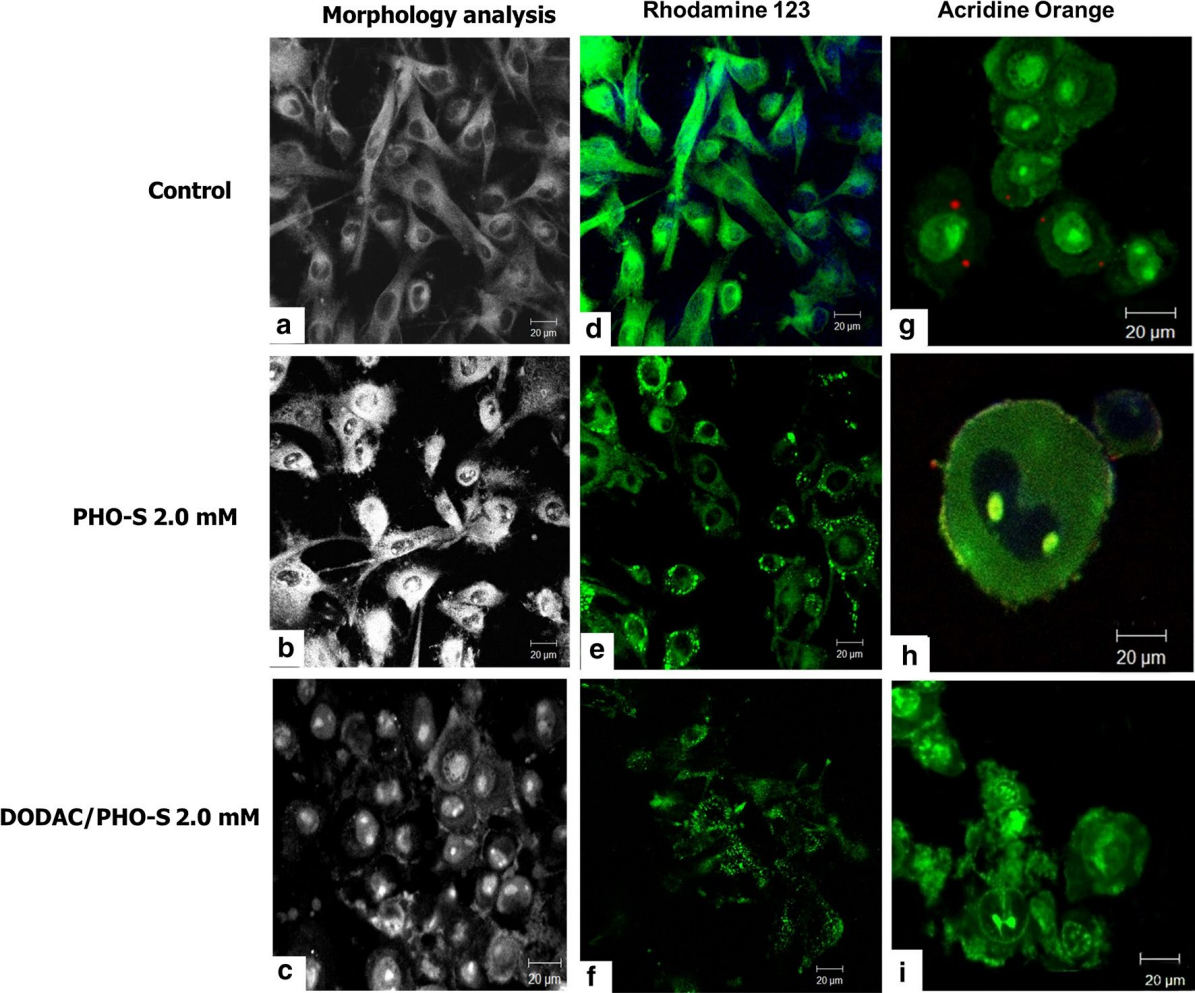
Photomicrographs of B16F10 murine melanoma cells after treatment with PHO-S, DODAC/PHO-S and DODAC. B16F10 cells after treatment, for a period of 6 h, with evaluation of morphology, mitochondrial electrical potential (ΔΨm) and lysosome integrity. Morphological analysis of the **(a)** control; **b** treatment with PHO-S (2.0 mM) and **c** DODAC/PHO-S (2.0 mM). Analysis of mitochondrial electrical potential with rhodamine 123 (green) of the **(d)** control; **e** treatment with PHO-S (2.0 mM) and **f** DODAC/PHO-S (2.0 mM). Loss of lysosomal integrity measured by acridine orange staining of the Lysosomal Integrity with acridine orange of the **(g)** Control; treatment with **(h)** PHO-S (2.0 mM) and **i** DODAC/PHO-S (2.0 mM). Photomicrographs represent results from three independent experiments

The cells of the control group presented fibroblast-like appearance, with cytoplasmic extensions and evident nucleus (Fig. 1a). After the treatment period, cells treated with PHO-S underwent morphological changes, such as cytoplasmic retraction (Fig. 1b). In contrast, treatment with liposomes culminated in a greater change in B16F10 cells, such as loss of its morphology, cytoplasmic retraction and formation of apoptotic bodies between adherent cells (Fig. 1c).

### Evaluation of mitochondrial electrical potential (ΔΨm) and lysosome integrity

It was possible to observe that the cells of the control group had a positive marking for rhodamine 123, demonstrating the large number of active mitochondria in these cells and homogeneously distributed through the cytoplasm (Fig. 1d). After treatment with PHO-S, in higher concentration, it was possible to observe that there was a reduction in the fluorescence emission and a modification in the morphology of the mitochondria in the cytoplasm of the cells. It was also possible to observe that mitochondria fused after treatment (Fig. 1e). Treatment with DODAC/PHO-S liposomes, in all concentrations, reduced fluorescence emission compared to isolated PHO-S, exhibiting low fluorescence emission and loss of cell morphology (Fig. 1f). Liposomes led to disorganisation and depolarisation of the mitochondria.

In the untreated B16F10 tumour cells, it was possible to indicate the nucleus of the cells and the organisation of the condensed (green) chromatin structure and few lysosomes in the cytoplasm (red) (Fig. 1g). The cells that were treated with PHO-S and DODAC/PHO-S (2.0 mM) liposomes did not present intact lysosomes (Fig. 1h, i). However, only treatment with the DODAC/PHO-S liposomes led to chromatin disruption and destruction of cells, at the evaluated time of treatment, as well as a significant decrease in cell density (Fig. 1i).

### Expression of pro-apoptotic proteins after treatment of B16F10 cells

**Fig. 2:**
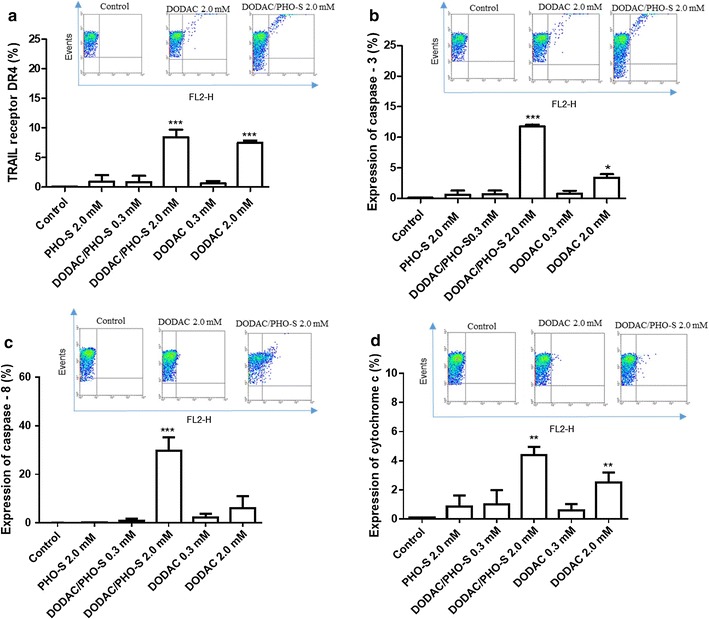
Analysis of protein expression on B16F10 murine melanoma cells. Analysis of expression of pro-apoptotic proteins quantified by flow cytometry, after 24 h of treatment with PHO-S, DODAC and DODAC/PHO-S. Analysis of expression of trail receptor DR4 (**a**); caspase 3 (**b**) and 9 (**c**) active and cytochrome c free (**d**) quantified by flow cytometry, after 24 h of treatment with PHO-S, DODAC and DODAC/PHO-S. Each value is the mean of at least three independent experiments in each group

The concentrations used in the treatment were those that showed the greatest effectiveness in inducing significant cytotoxicity in B16F10 cells. The expression of TRAIL-DR4 receptor increased significantly only in treatments with DODAC and DODAC/PHO-S 1:1 (2.0 mM) in B16F10 melanoma cells. The respective percentages were 7.5 ± 1.3 and 8.4 ± 0.4% (Fig. 2a).

Treatment with DODAC/PHO-S (2.0 mM) was effective in modulating the expression of active caspases 3 and 8, with mean values of 11.7 ± 0.3 and 29.8 ± 5.5%, respectively (Fig. 2b, c). The DODAC 2.0 mM also modulated the expression of caspase 3, with a significant increase in this treatment, with the percentage of 3.4 ± 0.6%, but to a smaller extent than the liposomal formulation (Fig. 2b).

The free cytochrome c in the cytoplasm was high in the treatments with DODAC/PHO-S 2.0 mM and DODAC 2.0 mM, the mean values presented are 4.4 ± 0.6 and 2.5 ± 0.7%, respectively (Fig. 2d).

## Discussion

Recently, the PHO-S was encapsulated in DODAC liposomes by our research group, aiming to maximize the antitumor effect and providing a greater availability of PHO-S in the tumour microenvironment [4].

The results of this study demonstrate DODAC/PHO-S liposomes have exhibited broad cytotoxic potential in B16F10 murine melanoma cells, with significantly greater proportions than treatment with PHO-S. This result demonstrates the efficacy of liposomal formulation DODAC/PHO-S to promote B16F10 cell death. Recently, these liposomes were evaluated on normal and did not promote significant cell death [4]. Studies have shown that the use of nanocarriers are promising drug delivery systems, presenting several advantages such as low skin irritation and increased protection of the encapsulated drug. The use of nanocarriers may also increase the penetration of anti-tumour drugs into the skin [10].

Cytotoxic compounds for tumour cells can lead to the decreased mitochondrial electrical potential (ΔmΨ) [11, 12]. Studies have shown that PHO-S has the ability to lead to the loss of ΔmΨ and morphological changes in Ehrlich’s tumour [1]. Therefore, treatment with the DODAC/PHO-S 2.0 mM liposomal formulation was more efficient in decreasing mitochondrial electrical potential at the same concentrations and treatment time than PHO-S. Corroborating our findings, other results showed that PHO-S could modify the distribution of mitochondria in the cytoplasm, leading to its fragmentation and aggregation [8]. Several studies have shown that mitochondrial dysfunctions represent a central process in the induction of apoptosis [11].

It was verified in our study, that after treatment with the PHO-S 2.0 mM it was not possible to verify intact lysosomes, probably due to its rupture, since it is evident in the more intense green coloration in the treated cells. However, treatment with the liposomal formulation DODAC/PHO-S (2.0 mM) was more efficient to promote morphological changes in the cells, without presenting intact lysosomes, at the same time of treatment and concentration as PHO-S.

Taking into consideration of the morphological, structural and energetic changes that the cells suffered after treatment with PHO-S and liposomal formulation, the data indicate that the liposomal formulation can lead the cells to activate mechanisms of programmed cell death, faster and efficient. The changes described after the treatments are like those occurring during the apoptotic process [12, 13]. Therefore, the proteins involved in the apoptotic caspase 3 and 8, DR4 receptor and free cytochrome c treatments were quantified after the treatments with PHO-S and DODAC/PHO-S liposomes.

Our results demonstrated that the liposomal formulation increased DR4 receptor expression and activated caspases 8 and 3, resulting in the release of cytochrome c in B16F10 tumour cells, when compared to treatment with PHO-S.

The increase of the active 8 and 3 caspases, TRAIL receptor-DR4 expression and the release of cytochrome c in B16F10 tumour cells, demonstrated the efficacy of the liposomal formulation in maximising the apoptotic potential mediated by PHO-S. Most of the apoptotic events are required to participate in the caspases, which are divided into initiators (8, 9 and 12) and executors (3, 6 and 7), where the latter are responsible for morphological changes in the nucleus and cytoskeleton [14]. The PHO-S induced apoptosis in several tumour cell lines, increasing the expression of pro-caspase 8 that can be classified as a caspase-8-like. Previous studies have shown that PHO-S induced cell death by apoptosis, with increased expression of pro-caspase 3 and the presence of tBid, indicating that the intrinsic pathway was recruited in response to activation of caspase 8 [1–3].

The data obtained prove that the use of DODAC as carrier can maximize the cytotoxic effects of PHO-S. This was demonstrated by the translocation of cytochrome c to the cytoplasm and activation of caspase-3 and 8. In B16F10 cells, the laser confocal microscopy analysis demonstrated the efficiency of DODAC/PHO-S liposomes in decreasing the mitochondrial electrical potential and generating morphological changes.

## Limitations

The results of mitochondrial electrical potential and lysosome integrity were measured by confocal microscopy. Thus, the quantification of results and bright field can be needed. Furthermore, western blot analysis of each apoptotic protein can be needed to confirm the fact.

## Abbreviations

PHO-S: DODAC Dioctadecyldimethylammonium Chloride
ATCC: American Type Culture Collection
ΔmΨ: mitochondrial electrical potential
